# Bis(2,2′-bipyridine)[1,9-bis(diphenylphos­phanyl)-1,2,3,4,6,7,8,9-octahydropyrim­ido[1,2-*a*]pyrimidin-5-ium]ruthenium(II) hexa­fluorido­phosphate dibromide di­chloro­methane disolvate monohydrate

**DOI:** 10.1107/S1600536813029462

**Published:** 2013-11-06

**Authors:** Congcong Shang, Laure Vendier, Pierre Sutra, Alain Igau

**Affiliations:** a Laboratoire de Chimie de Coordination, UPR-CNRS 8241, 205, route de Narbonne, 31077 Toulouse cedex, France

## Abstract

In the cation of the title complex, [Ru(C_31_H_32_N_3_P_2_)(C_10_H_8_N_2_)_2_](PF_6_)(Br)_2_·2CH_2_Cl_2_·H_2_O, the ruthenium ion is coordinated in a distorted octa­hedral geometry by two 2,2′-bi­pyridine (bpy) ligands and a chelating cationic *N*-di­phenyl­phosphino-1,3,4,6,7,8-hexa­hydro-2-pyrimido[1,2-*a*]pyrimidine [(PPh_2_)_2_-hpp] ligand. The tricationic charge of the complex is balanced by two bromide and one hexa­fluorido­phosphate counter-anions. The compound crystallized with two mol­ecules of di­chloro­methane (one of which is equally disordered about a Cl atom) and a water mol­ecule. In the crystal, one of the Br anions bridges two water mol­ecules *via* O—H⋯Br hydrogen bonds, forming a centrosymmetric diamond-shaped *R* ^4^_2_(8) motif. The cation and anions and the solvent mol­ecules are linked *via* C—H⋯F, C—H⋯Br, C—H⋯Cl and C—H⋯O hydrogen bonds, forming a three-dimensional network.

## Related literature

For the synthesis of the precursor, [Ru(bpy)_2_(Ph_2_PH)_2_](PF_6_)_2_, see: Sullivan *et al.* (1978 ▶).
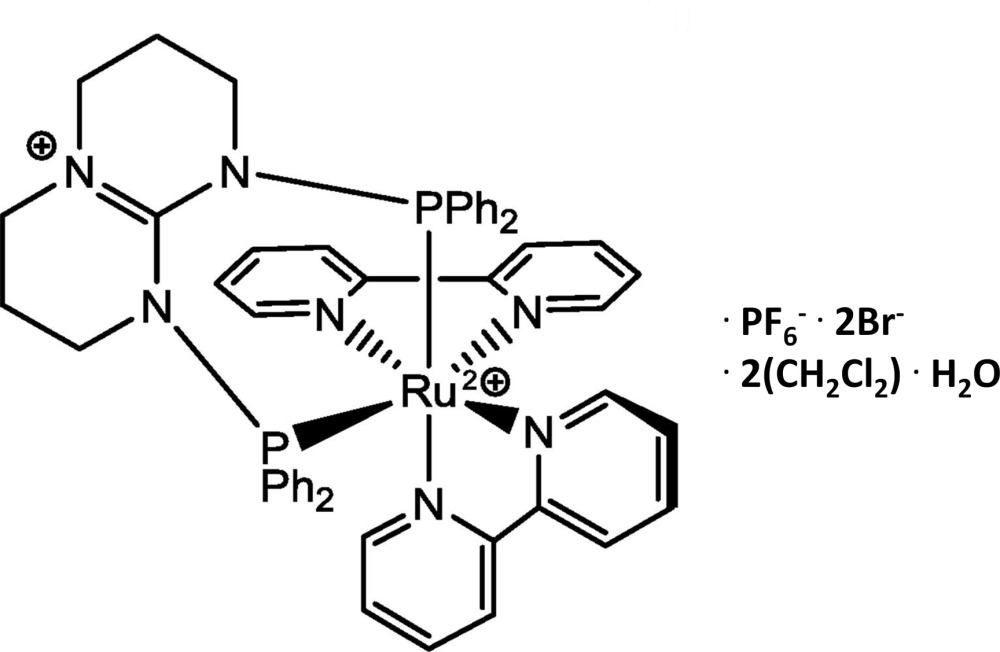


## Experimental

### 

#### Crystal data

[Ru(C_31_H_32_N_3_P_2_)(C_10_H_8_N_2_)_2_](PF_6_)(Br)_2_·2CH_2_Cl_2_·H_2_O
*M* *_r_* = 1414.63
Monoclinic, 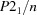

*a* = 16.1770 (4) Å
*b* = 20.9840 (5) Å
*c* = 16.6730 (4) Å
β = 96.654 (2)°
*V* = 5621.7 (2) Å^3^
*Z* = 4
Mo *K*α radiation
μ = 2.04 mm^−1^
*T* = 100 K
0.18 × 0.05 × 0.04 mm

#### Data collection

Agilent Xcalibur (Eos, Gemini ultra) diffractometer
Absorption correction: multi-scan (*CrysAlis PRO*; Agilent, 2012 ▶) *T*_min_ = 0.964, *T*_max_ = 1.0
44290 measured reflections
10285 independent reflections
7429 reflections with *I* > 2σ(*I*)
*R*_int_ = 0.079

#### Refinement

*R*[*F* ^2^ > 2σ(*F* ^2^)] = 0.055
*wR*(*F* ^2^) = 0.131
*S* = 1.04
10285 reflections
718 parameters
3 restraints
H atoms treated by a mixture of independent and constrained refinement
Δρ_max_ = 1.72 e Å^−3^
Δρ_min_ = −1.69 e Å^−3^

### 

Data collection: *CrysAlis PRO* (Agilent, 2012 ▶); cell refinement: *CrysAlis PRO*; data reduction: *CrysAlis PRO*; program(s) used to solve structure: *SIR92* (Altomare *et al.*, 1994 ▶); program(s) used to refine structure: *SHELXL97* (Sheldrick, 2008 ▶); molecular graphics: *PLATON* (Spek, 2009 ▶); software used to prepare material for publication: *WinGX* publication routines (Farrugia, 2012 ▶).

## Supplementary Material

Crystal structure: contains datablock(s) global, I. DOI: 10.1107/S1600536813029462/su2644sup1.cif

Structure factors: contains datablock(s) I. DOI: 10.1107/S1600536813029462/su2644Isup2.hkl

Supplementary material file. DOI: 10.1107/S1600536813029462/su2644Isup3.cdx

968714

Additional supplementary materials:  crystallographic information; 3D view; checkCIF report

## Figures and Tables

**Table 1:** Hydrogen-bond geometry (Å, °)

*D*—H⋯*A*	*D*—H	H⋯*A*	*D*⋯*A*	*D*—H⋯*A*
O1—H1*A*⋯Br1^i^	0.99 (5)	2.31 (5)	3.300 (5)	173 (3)
O1—H1*B*⋯Br1^ii^	1.00 (4)	2.35 (4)	3.345 (5)	178 (6)
C4—H4*A*⋯Br2^iii^	0.99	2.90	3.651 (5)	133
C6—H6*A*⋯Cl7	0.99	2.82	3.738 (8)	155
C6—H6*B*⋯O1^iv^	0.99	2.52	3.358 (8)	142
C9—H9⋯O1^v^	0.95	2.40	3.208 (8)	142
C10—H10⋯Cl3^vi^	0.95	2.72	3.316 (8)	122
C100—H10*A*⋯Br1^vii^	0.99	2.92	3.618 (11)	128
C106—H10*C*⋯Cl1	0.99	2.65	3.54 (2)	150
C106—H10*D*⋯O1^iv^	0.99	2.51	3.464 (16)	162
C11—H11⋯F3^vii^	0.95	2.35	3.209 (7)	150
C17—H17⋯F5^iii^	0.95	2.25	3.056 (6)	142
C21—H21⋯Br1^viii^	0.95	2.91	3.787 (5)	154
C24—H24⋯Br1^viii^	0.95	2.91	3.835 (5)	164
C25—H25⋯F4^ix^	0.95	2.54	3.453 (6)	160
C31—H31⋯Cl3^x^	0.95	2.76	3.566 (7)	143
C42—H42⋯F3^iii^	0.95	2.41	3.319 (7)	160

Symmetry codes: (i) 
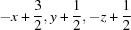
; (ii) 
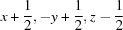
; (iii) 
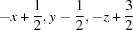
; (iv) 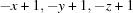; (v) 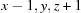; (vi) 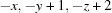; (vii) 
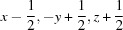
; (viii) 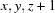; (ix) 
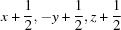
; (x) 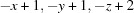.

**Fig. 1.**
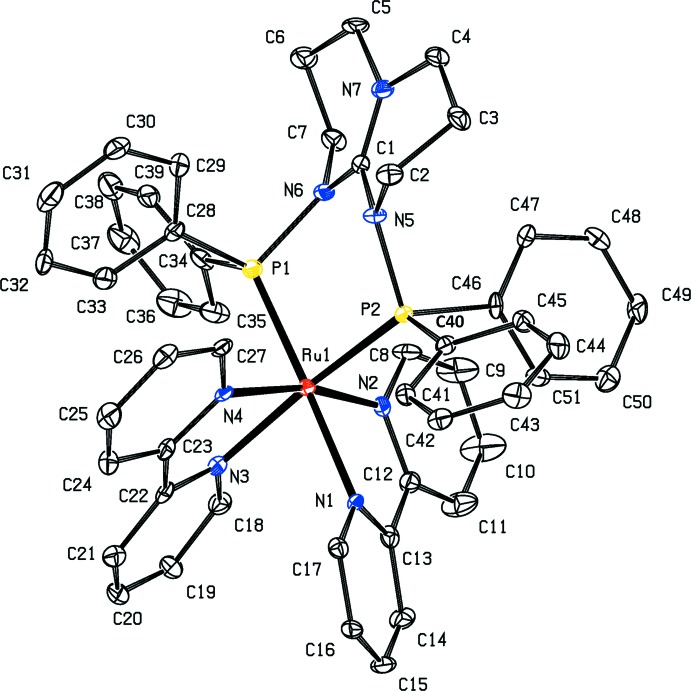
The molecule structure of the title cation, with the atom labelling. The displacement ellipsoids are drawn at the 50% probability level. The H atoms and the anions and solvent molecules of crystallization have been omitted for clarity.

## supplementary crystallographic information

### 1. Comment

In our efforts to functionnalize bisphosphino bisbipyridine ruthenium complexes we investigated the reaction of 1,2-dibromoethane with [Ru(bpy)_2_(PHPh_2_)]^2+^ in the presence of a base in order to form the ruthenium complex incorporating a chelating diphenylphosphino ethane ligand. This compound was previously obtained by (Sullivan *et al.*, 1978) through coordination of the corresponding diphenylphosphino ethane (dppe) on the [Ru(bpy)_2_Cl_2_]^2+^ complex with a silver salt as a choride abstractor. The addition of an excess of BrCH_2_CH_2_Br into an acetonitrile solution of the precursor complex [Ru(bpy)_2_(PH*R*_2_)]^2+^ in the presence of 4 equivalents of 1,3,4,6,7,8-hexahydro-2-pyrimido[1,2-*a*]pyrimidine (H-hpp) gave the title compound as the major component.

In the cation of the title compound, Fig. 1, the ruthenium ion is coordinated in a distorted octahedral geometry to two 2,2'-bipyridine (bpy) ligands and a chelating cationic *N*-diphenylphosphino-1,3,4,6,7,8-hexahydro-2-pyrimido[1,2-*a*]pyrimidine [(PPh_2_)_2_-hpp] ligand. The tricationic charge of the complex is balanced by two bromide and one hexafluorophosphate counter anions. The compound crystallized as a dichloromethane disolvate and monohydrate.

In the crystal, one Br anion bridges two water molecules via O-H···Br hydrogen bonds forming a centrosymmetric diamond shaped R^4^_2_(8) motif (Table 1). The cation and anions and the solvent molecules are linked via C-H···F, C-H···Br, C-H···Cl and C-H···O hydrogen bonds forming a three-dimensional structure (Table 1).

Instead of the expected insertion of the ethane fragment as a unit bridging the two coordinated diphenylphosphino ligands, we observed the formal insertion of a guanidinate fragment linking the two phosphorus atoms. This resulting complex was characterized in ^31^P NMR by a sharp singlet at 118.1 p.p.m. associated to the functionalized Ph_2_P—N group. The analysis of the crude mixture by ^1^H NMR revealed the formation of bromoethylene in the course of the reaction. The formation of this by-product implies a dehydrobromation of the initial dibromoethane reagent and the release of HBr probably trapped by an H-hpp molecule to form the corresponding guanidinium bromide. The latter guanidinium may act as a potential reagent in the formation of the title complex. The complete understanding of the particular reactivity of the [Ru(bpy)_2_(PH*R*_2_)]^2+^ complex with dibromoethane and H-hpp requires further studies that are currently under progress.

### 2. Experimental

All manipulations were carried out in dry solvents and under dry argon atmosphere. The precursor complex [Ru(bpy)_2_(Ph_2_PH)_2_](PF_6_)_2_ was prepared according to a previously described procedure (Sullivan *et al.*, 1978). 1,3,4,6,7,8-Hexahydro-2*H*-pyrimido[1,2-*a*]pyrimidine (H-hpp) (Sigma-Aldrich) was used without further purification. This precursor complex [Ru(bpy)_2_(Ph_2_PH)_2_](PF_6_)_2_ (50 mg, 0.046 mmol) was dissolved in acetonitrile (3 ml) and transferred onto 4 equivalents of H-hpp (26 mg, 0.18 mmol) using a standard Schlenk-line and cannula techniques under a dry argon atmosphere. After further addition of a ten-fold excess of BrCH_2_CH_2_Br (40 µ*L*, 0.46 mmol) under argon, the reaction mixture was stirred for 30 min at room temperature. Addition of the crude mixture to 50 ml of Et_2_O induced precipitation of a light yellow/orange solid which was isolated by filtration and dried under vacuum. Dissolution of 15 mg of this solid in 0.5 ml of dry CH_2_Cl_2_ followed by careful addition of 3 ml of diethylether as a non-solvent afforded, after 3 days at room temperature, yellow needle-like crystals of the title compound, suitable for X-ray diffraction analysis.

### 3. Refinement

The water H atoms were located in a difference Fourier map and freely refined. The C-bound H atoms were positioned geometrically (C—H = 0.95 - 1.00 Å) and refined as riding on their parent atoms, with U_iso_(H) = 1.2U_eq_(C). One of the dichloromethane molecules is disordered about a Cl atom, viz. atom Cl3 (occupancy 0.5).

### Crystal data

**Table:**

[Ru(C_31_H_32_N_3_P_2_)(C_10_H_8_N_2_)_2_](PF_6_)Br_2_·2CH_2_Cl_2_·H_2_O	*F*(000) = 2840
*M**_r_* = 1414.63	*D*_x_ = 1.671 Mg m^−^^3^
Monoclinic, *P*2_1_/*n*	Mo *K*α radiation, λ = 0.71073 Å
Hall symbol: -P 2yn	Cell parameters from 6399 reflections
*a* = 16.1770 (4) Å	θ = 2.9–29.3°
*b* = 20.9840 (5) Å	µ = 2.04 mm^−^^1^
*c* = 16.6730 (4) Å	*T* = 100 K
β = 96.654 (2)°	Needle, yellow
*V* = 5621.7 (2) Å^3^	0.18 × 0.05 × 0.04 mm
*Z* = 4	

### Data collection

**Table:**

Agilent Xcalibur (Eos, Gemini ultra) diffractometer	10285 independent reflections
Graphite monochromator	7429 reflections with *I* > 2σ(*I*)
Detector resolution: 16.1978 pixels mm^-1^	*R*_int_ = 0.079
ω scans	θ_max_ = 25.4°, θ_min_ = 3.1°
Absorption correction: multi-scan (*CrysAlis PRO*; Agilent, 2012)	*h* = −19→19
*T*_min_ = 0.964, *T*_max_ = 1.0	*k* = −25→25
44290 measured reflections	*l* = −20→20

### Refinement

**Table:**

Refinement on *F*^2^	Primary atom site location: structure-invariant direct methods
Least-squares matrix: full	Secondary atom site location: difference Fourier map
*R*[*F*^2^ > 2σ(*F*^2^)] = 0.055	Hydrogen site location: inferred from neighbouring sites
*wR*(*F*^2^) = 0.131	H atoms treated by a mixture of independent and constrained refinement
*S* = 1.04	*w* = 1/[σ^2^(*F*_o_^2^) + (0.0435*P*)^2^ + 24.738*P*] where *P* = (*F*_o_^2^ + 2*F*_c_^2^)/3
10285 reflections	(Δ/σ)_max_ = 0.003
718 parameters	Δρ_max_ = 1.72 e Å^−^^3^
3 restraints	Δρ_min_ = −1.69 e Å^−^^3^

### Special details

**Table:**

Geometry. Bond distances, angles etc. have been calculated using the rounded fractional coordinates. All su's are estimated from the variances of the (full) variance-covariance matrix. The cell esds are taken into account in the estimation of distances, angles and torsion angles
Refinement. Refinement of *F*^2^ against ALL reflections. The weighted *R*-factor *wR* and goodness of fit *S* are based on *F*^2^, conventional *R*-factors *R* are based on *F*, with *F* set to zero for negative *F*^2^. The threshold expression of *F*^2^ > σ(*F*^2^) is used only for calculating *R*-factors(gt) *etc*. and is not relevant to the choice of reflections for refinement. *R*-factors based on *F*^2^ are statistically about twice as large as those based on *F*, and *R*- factors based on ALL data will be even larger.

### Fractional atomic coordinates and isotropic or equivalent isotropic displacement parameters (Å^2^)

**Table:**

	*x*	*y*	*z*	*U*_iso_*/*U*_eq_	Occ. (<1)
Ru1	0.16075 (2)	0.18750 (2)	1.06626 (2)	0.0086 (1)	
P1	0.22497 (8)	0.28461 (7)	1.07860 (8)	0.0112 (4)	
P2	0.13101 (8)	0.20991 (6)	0.93213 (8)	0.0087 (4)	
N1	0.0950 (2)	0.0986 (2)	1.0652 (2)	0.0092 (12)	
N2	0.0410 (3)	0.2163 (2)	1.0855 (3)	0.0118 (12)	
N3	0.1887 (3)	0.1558 (2)	1.1890 (3)	0.0129 (12)	
N4	0.2769 (2)	0.1431 (2)	1.0649 (3)	0.0101 (12)	
N5	0.1981 (3)	0.26713 (19)	0.8966 (3)	0.0100 (12)	
N6	0.1841 (3)	0.3392 (2)	1.0031 (3)	0.0114 (12)	
N7	0.2028 (3)	0.3769 (2)	0.8750 (3)	0.0126 (12)	
C1	0.1953 (3)	0.3287 (2)	0.9243 (3)	0.0108 (17)	
C2	0.2373 (3)	0.2608 (3)	0.8204 (3)	0.0149 (17)	
C3	0.1907 (3)	0.3027 (3)	0.7566 (3)	0.0167 (17)	
C4	0.1973 (3)	0.3714 (3)	0.7859 (3)	0.0166 (17)	
C5	0.2185 (4)	0.4437 (2)	0.9020 (3)	0.0183 (17)	
C6	0.2187 (4)	0.4516 (3)	0.9929 (3)	0.0196 (17)	
C7	0.1565 (3)	0.4053 (2)	1.0204 (3)	0.0151 (17)	
C8	0.0097 (3)	0.2764 (3)	1.0803 (3)	0.0169 (17)	
C9	−0.0705 (4)	0.2905 (3)	1.0931 (4)	0.029 (2)	
C10	−0.1216 (4)	0.2419 (3)	1.1140 (5)	0.035 (2)	
C11	−0.0916 (4)	0.1801 (3)	1.1182 (4)	0.0271 (19)	
C12	−0.0108 (3)	0.1683 (3)	1.1030 (3)	0.0134 (17)	
C13	0.0222 (3)	0.1033 (3)	1.0976 (3)	0.0120 (17)	
C14	−0.0164 (3)	0.0498 (3)	1.1255 (3)	0.0167 (17)	
C15	0.0178 (3)	−0.0095 (3)	1.1163 (3)	0.0152 (17)	
C16	0.0899 (3)	−0.0148 (2)	1.0796 (3)	0.0129 (17)	
C17	0.1272 (3)	0.0404 (2)	1.0555 (3)	0.0116 (17)	
C18	0.1387 (3)	0.1613 (3)	1.2482 (3)	0.0152 (17)	
C19	0.1509 (3)	0.1268 (3)	1.3195 (3)	0.0175 (17)	
C20	0.2170 (3)	0.0845 (3)	1.3298 (3)	0.0214 (17)	
C21	0.2690 (3)	0.0778 (3)	1.2701 (3)	0.0176 (17)	
C22	0.2534 (3)	0.1136 (3)	1.1996 (3)	0.0144 (17)	
C23	0.3046 (3)	0.1093 (3)	1.1319 (3)	0.0137 (17)	
C24	0.3792 (3)	0.0758 (3)	1.1392 (3)	0.0175 (17)	
C25	0.4279 (3)	0.0772 (3)	1.0759 (4)	0.0199 (19)	
C26	0.4020 (3)	0.1139 (3)	1.0089 (3)	0.0175 (17)	
C27	0.3265 (3)	0.1452 (2)	1.0050 (3)	0.0127 (17)	
C28	0.3367 (3)	0.2821 (3)	1.0734 (3)	0.0135 (17)	
C29	0.3789 (3)	0.3049 (3)	1.0110 (3)	0.0171 (17)	
C30	0.4642 (3)	0.2938 (3)	1.0125 (4)	0.0217 (17)	
C31	0.5067 (4)	0.2608 (3)	1.0762 (4)	0.0251 (19)	
C32	0.4660 (3)	0.2390 (3)	1.1396 (4)	0.0207 (17)	
C33	0.3824 (4)	0.2502 (3)	1.1390 (3)	0.0180 (17)	
C34	0.2233 (4)	0.3333 (3)	1.1694 (3)	0.0161 (17)	
C35	0.1644 (4)	0.3244 (3)	1.2224 (3)	0.0206 (17)	
C36	0.1653 (4)	0.3621 (3)	1.2915 (4)	0.0261 (19)	
C37	0.2240 (4)	0.4105 (3)	1.3059 (4)	0.0269 (19)	
C38	0.2830 (4)	0.4191 (3)	1.2531 (3)	0.0239 (19)	
C39	0.2835 (4)	0.3807 (3)	1.1861 (3)	0.0181 (17)	
C40	0.1329 (3)	0.1473 (2)	0.8561 (3)	0.0113 (17)	
C41	0.1784 (3)	0.0913 (3)	0.8701 (3)	0.0126 (17)	
C42	0.1763 (3)	0.0439 (3)	0.8122 (3)	0.0140 (17)	
C43	0.1275 (3)	0.0513 (3)	0.7391 (3)	0.0185 (17)	
C44	0.0819 (4)	0.1069 (3)	0.7233 (3)	0.0191 (17)	
C45	0.0841 (3)	0.1541 (3)	0.7813 (3)	0.0145 (17)	
C46	0.0257 (3)	0.2402 (2)	0.9035 (3)	0.0110 (17)	
C47	0.0047 (3)	0.3013 (3)	0.8753 (3)	0.0146 (17)	
C48	−0.0788 (3)	0.3179 (3)	0.8561 (3)	0.0171 (17)	
C49	−0.1411 (3)	0.2750 (3)	0.8654 (3)	0.0191 (17)	
C50	−0.1211 (3)	0.2139 (3)	0.8935 (3)	0.0190 (17)	
C51	−0.0386 (3)	0.1967 (3)	0.9116 (3)	0.0147 (17)	
P4	0.17252 (9)	0.41418 (7)	0.54217 (9)	0.0184 (5)	
F1	0.2120 (3)	0.3586 (2)	0.4949 (2)	0.0469 (17)	
F2	0.1156 (2)	0.36512 (19)	0.5839 (2)	0.0416 (14)	
F3	0.2447 (2)	0.40650 (16)	0.6163 (2)	0.0260 (11)	
F4	0.1330 (2)	0.47123 (18)	0.5888 (2)	0.0353 (12)	
F5	0.2283 (2)	0.46563 (19)	0.5014 (2)	0.0386 (12)	
F6	0.1007 (2)	0.42265 (18)	0.4683 (2)	0.0349 (12)	
Cl3	0.2960 (2)	0.70831 (18)	0.9915 (3)	0.0480 (14)	0.500
Cl4	0.36669 (13)	0.57601 (10)	0.97660 (11)	0.0510 (7)	
Cl7	0.4359 (2)	0.4762 (2)	1.0914 (4)	0.0715 (19)	0.500
C105	0.4601 (11)	0.5292 (12)	1.0141 (11)	0.081 (8)	0.500
C106	0.2731 (9)	0.6301 (7)	0.9517 (14)	0.067 (7)	0.500
Cl1	0.1583 (2)	0.60233 (13)	0.7607 (3)	0.172 (3)	
Cl2	0.04304 (17)	0.69559 (13)	0.68604 (15)	0.0795 (10)	
C100	0.0559 (8)	0.6172 (5)	0.7220 (7)	0.096 (5)	
Br1	0.48199 (5)	0.01025 (4)	0.34126 (4)	0.0422 (3)	
Br2	0.07640 (4)	0.88384 (3)	0.67696 (4)	0.0315 (2)	
O1	0.9184 (3)	0.4281 (2)	0.0087 (3)	0.0417 (19)	
H2A	0.23500	0.21580	0.80230	0.0180*	
H2B	0.29640	0.27400	0.82970	0.0180*	
H3A	0.13150	0.28960	0.74730	0.0200*	
H3B	0.21510	0.29850	0.70510	0.0200*	
H4A	0.24730	0.39110	0.76730	0.0200*	
H4B	0.14800	0.39540	0.76140	0.0200*	
H5A	0.17500	0.47160	0.87380	0.0220*	
H5B	0.27290	0.45770	0.88650	0.0220*	
H6A	0.27490	0.44280	1.02090	0.0240*	
H6B	0.20320	0.49580	1.00570	0.0240*	
H7A	0.15330	0.41040	1.07900	0.0180*	
H7B	0.10060	0.41350	0.99140	0.0180*	
H8	0.04490	0.31010	1.06720	0.0200*	
H9	−0.09070	0.33300	1.08770	0.0350*	
H10	−0.17660	0.25090	1.12540	0.0420*	
H11	−0.12620	0.14610	1.13150	0.0320*	
H14	−0.06600	0.05400	1.15050	0.0200*	
H15	−0.00790	−0.04650	1.13520	0.0190*	
H16	0.11340	−0.05540	1.07110	0.0160*	
H17	0.17730	0.03690	1.03120	0.0140*	
H18	0.09310	0.19010	1.24060	0.0180*	
H19	0.11490	0.13200	1.36020	0.0210*	
H20	0.22660	0.06000	1.37790	0.0260*	
H21	0.31470	0.04910	1.27710	0.0210*	
H24	0.39670	0.05230	1.18690	0.0210*	
H25	0.47810	0.05340	1.07860	0.0240*	
H26	0.43560	0.11760	0.96610	0.0210*	
H27	0.30860	0.16940	0.95800	0.0150*	
H29	0.34970	0.32790	0.96760	0.0210*	
H30	0.49290	0.30890	0.96970	0.0260*	
H31	0.56460	0.25300	1.07660	0.0300*	
H32	0.49570	0.21640	1.18310	0.0250*	
H33	0.35490	0.23640	1.18320	0.0220*	
H35	0.12300	0.29240	1.21170	0.0250*	
H36	0.12590	0.35480	1.32860	0.0320*	
H37	0.22360	0.43740	1.35170	0.0320*	
H38	0.32360	0.45180	1.26300	0.0290*	
H39	0.32510	0.38660	1.15090	0.0220*	
H41	0.21160	0.08550	0.92040	0.0150*	
H42	0.20830	0.00620	0.82280	0.0170*	
H43	0.12510	0.01840	0.69970	0.0220*	
H44	0.04930	0.11250	0.67260	0.0230*	
H45	0.05210	0.19170	0.77040	0.0180*	
H47	0.04720	0.33160	0.86920	0.0170*	
H48	−0.09270	0.35950	0.83640	0.0200*	
H49	−0.19770	0.28710	0.85270	0.0230*	
H50	−0.16390	0.18410	0.90020	0.0230*	
H51	−0.02520	0.15470	0.92980	0.0170*	
H10C	0.25880	0.63250	0.89240	0.0800*	0.500
H10D	0.22460	0.61230	0.97530	0.0800*	0.500
H998	0.48020	0.50480	0.97180	0.0990*	0.500
H999	0.50480	0.55660	1.03640	0.0990*	0.500
H10A	0.01920	0.61000	0.76470	0.1140*	
H10B	0.03920	0.58710	0.67740	0.1140*	
H1A	0.947 (4)	0.450 (3)	0.0569 (19)	0.0500*	
H1B	0.936 (4)	0.446 (3)	−0.0423 (17)	0.0500*	

### Atomic displacement parameters (Å^2^)

**Table:**

	*U*^11^	*U*^22^	*U*^33^	*U*^12^	*U*^13^	*U*^23^
Ru1	0.0088 (2)	0.0082 (2)	0.0089 (2)	−0.0012 (2)	0.0011 (2)	0.0006 (2)
P1	0.0142 (7)	0.0113 (7)	0.0080 (7)	−0.0026 (6)	0.0006 (5)	0.0000 (6)
P2	0.0085 (6)	0.0080 (6)	0.0096 (7)	−0.0009 (5)	0.0009 (5)	0.0003 (5)
N1	0.009 (2)	0.009 (2)	0.009 (2)	−0.0032 (18)	−0.0009 (17)	−0.0001 (18)
N2	0.010 (2)	0.015 (2)	0.010 (2)	−0.0014 (19)	−0.0011 (17)	0.0031 (19)
N3	0.012 (2)	0.011 (2)	0.015 (2)	−0.003 (2)	−0.0016 (18)	0.0007 (19)
N4	0.009 (2)	0.010 (2)	0.011 (2)	−0.0048 (18)	0.0005 (17)	0.0010 (18)
N5	0.012 (2)	0.007 (2)	0.012 (2)	−0.0003 (18)	0.0053 (18)	0.0006 (18)
N6	0.014 (2)	0.008 (2)	0.012 (2)	−0.0022 (19)	0.0005 (18)	0.0009 (18)
N7	0.013 (2)	0.012 (2)	0.013 (2)	−0.0024 (19)	0.0030 (18)	0.0016 (19)
C1	0.010 (3)	0.012 (3)	0.010 (3)	−0.002 (2)	0.000 (2)	0.001 (2)
C2	0.015 (3)	0.015 (3)	0.016 (3)	0.000 (2)	0.008 (2)	0.000 (2)
C3	0.019 (3)	0.021 (3)	0.010 (3)	0.001 (2)	0.001 (2)	0.004 (2)
C4	0.016 (3)	0.020 (3)	0.014 (3)	−0.002 (2)	0.003 (2)	0.007 (2)
C5	0.027 (3)	0.007 (3)	0.020 (3)	−0.006 (2)	−0.001 (2)	0.003 (2)
C6	0.027 (3)	0.011 (3)	0.020 (3)	−0.003 (3)	−0.001 (3)	0.000 (2)
C7	0.022 (3)	0.010 (3)	0.013 (3)	0.002 (2)	0.001 (2)	−0.001 (2)
C8	0.019 (3)	0.011 (3)	0.022 (3)	−0.001 (2)	0.008 (2)	0.006 (2)
C9	0.026 (3)	0.013 (3)	0.053 (5)	0.010 (3)	0.023 (3)	0.005 (3)
C10	0.024 (3)	0.022 (3)	0.065 (5)	0.008 (3)	0.030 (3)	0.006 (3)
C11	0.022 (3)	0.023 (3)	0.039 (4)	−0.003 (3)	0.015 (3)	0.003 (3)
C12	0.013 (3)	0.015 (3)	0.012 (3)	−0.002 (2)	0.001 (2)	0.000 (2)
C13	0.012 (3)	0.017 (3)	0.007 (3)	0.000 (2)	0.001 (2)	0.000 (2)
C14	0.016 (3)	0.021 (3)	0.014 (3)	−0.006 (3)	0.006 (2)	0.001 (2)
C15	0.015 (3)	0.011 (3)	0.020 (3)	−0.003 (2)	0.004 (2)	0.004 (2)
C16	0.016 (3)	0.007 (3)	0.015 (3)	0.000 (2)	−0.001 (2)	0.001 (2)
C17	0.011 (3)	0.014 (3)	0.010 (3)	0.001 (2)	0.002 (2)	0.001 (2)
C18	0.015 (3)	0.018 (3)	0.013 (3)	−0.004 (2)	0.003 (2)	−0.001 (2)
C19	0.020 (3)	0.021 (3)	0.011 (3)	−0.003 (3)	0.000 (2)	0.004 (2)
C20	0.020 (3)	0.028 (3)	0.015 (3)	−0.007 (3)	−0.003 (2)	0.011 (3)
C21	0.016 (3)	0.014 (3)	0.022 (3)	−0.002 (2)	−0.001 (2)	0.003 (2)
C22	0.013 (3)	0.014 (3)	0.015 (3)	−0.007 (2)	−0.003 (2)	−0.002 (2)
C23	0.012 (3)	0.013 (3)	0.015 (3)	−0.004 (2)	−0.003 (2)	−0.002 (2)
C24	0.016 (3)	0.018 (3)	0.017 (3)	−0.002 (2)	−0.004 (2)	0.001 (2)
C25	0.010 (3)	0.015 (3)	0.034 (4)	0.005 (2)	0.000 (2)	0.001 (3)
C26	0.014 (3)	0.019 (3)	0.020 (3)	−0.005 (2)	0.004 (2)	−0.003 (2)
C27	0.013 (3)	0.011 (3)	0.014 (3)	−0.006 (2)	0.001 (2)	−0.001 (2)
C28	0.014 (3)	0.012 (3)	0.014 (3)	−0.005 (2)	−0.001 (2)	−0.001 (2)
C29	0.016 (3)	0.017 (3)	0.017 (3)	−0.003 (2)	−0.004 (2)	0.001 (2)
C30	0.015 (3)	0.020 (3)	0.030 (3)	−0.006 (3)	0.002 (3)	0.005 (3)
C31	0.016 (3)	0.019 (3)	0.039 (4)	−0.004 (3)	−0.002 (3)	−0.006 (3)
C32	0.016 (3)	0.017 (3)	0.026 (3)	−0.004 (3)	−0.011 (3)	0.001 (3)
C33	0.025 (3)	0.016 (3)	0.012 (3)	−0.007 (3)	−0.002 (2)	−0.001 (2)
C34	0.026 (3)	0.011 (3)	0.010 (3)	0.001 (2)	−0.003 (2)	0.002 (2)
C35	0.030 (3)	0.015 (3)	0.017 (3)	−0.002 (3)	0.004 (2)	0.003 (2)
C36	0.040 (4)	0.024 (3)	0.016 (3)	0.002 (3)	0.011 (3)	−0.001 (3)
C37	0.044 (4)	0.021 (3)	0.015 (3)	0.004 (3)	0.000 (3)	−0.002 (3)
C38	0.033 (4)	0.016 (3)	0.020 (3)	0.001 (3)	−0.008 (3)	0.000 (3)
C39	0.024 (3)	0.018 (3)	0.011 (3)	−0.005 (3)	−0.003 (2)	0.002 (2)
C40	0.013 (3)	0.012 (3)	0.009 (3)	−0.003 (2)	0.002 (2)	−0.001 (2)
C41	0.010 (3)	0.018 (3)	0.010 (3)	0.000 (2)	0.002 (2)	0.002 (2)
C42	0.010 (3)	0.012 (3)	0.021 (3)	0.002 (2)	0.006 (2)	0.000 (2)
C43	0.026 (3)	0.015 (3)	0.015 (3)	0.000 (3)	0.005 (2)	−0.005 (2)
C44	0.025 (3)	0.021 (3)	0.010 (3)	−0.003 (3)	−0.003 (2)	−0.002 (2)
C45	0.015 (3)	0.013 (3)	0.015 (3)	0.001 (2)	0.000 (2)	0.001 (2)
C46	0.010 (3)	0.014 (3)	0.008 (3)	0.003 (2)	−0.003 (2)	−0.006 (2)
C47	0.011 (3)	0.017 (3)	0.015 (3)	−0.004 (2)	−0.002 (2)	0.000 (2)
C48	0.022 (3)	0.015 (3)	0.014 (3)	0.004 (3)	0.001 (2)	0.005 (2)
C49	0.007 (3)	0.027 (3)	0.023 (3)	0.005 (3)	0.000 (2)	0.001 (3)
C50	0.018 (3)	0.021 (3)	0.018 (3)	−0.003 (3)	0.002 (2)	−0.003 (3)
C51	0.019 (3)	0.012 (3)	0.013 (3)	−0.002 (2)	0.002 (2)	0.002 (2)
P4	0.0195 (8)	0.0192 (8)	0.0169 (8)	−0.0051 (7)	0.0034 (6)	0.0010 (6)
F1	0.049 (3)	0.047 (3)	0.043 (3)	0.017 (2)	−0.002 (2)	−0.023 (2)
F2	0.036 (2)	0.043 (2)	0.046 (3)	−0.0156 (19)	0.0063 (19)	0.017 (2)
F3	0.0264 (18)	0.0282 (19)	0.0229 (19)	0.0014 (16)	0.0006 (15)	−0.0011 (16)
F4	0.033 (2)	0.034 (2)	0.039 (2)	0.0078 (18)	0.0045 (17)	−0.0063 (18)
F5	0.034 (2)	0.049 (2)	0.033 (2)	−0.0179 (19)	0.0050 (17)	0.0101 (19)
F6	0.032 (2)	0.043 (2)	0.027 (2)	−0.0096 (18)	−0.0079 (16)	0.0055 (18)
Cl3	0.036 (2)	0.044 (2)	0.067 (3)	0.0024 (17)	0.0193 (19)	0.014 (2)
Cl4	0.0671 (13)	0.0561 (13)	0.0310 (10)	−0.0170 (11)	0.0108 (9)	−0.0011 (9)
Cl7	0.029 (2)	0.039 (2)	0.141 (5)	−0.0029 (18)	−0.013 (2)	0.029 (3)
C105	0.047 (11)	0.16 (2)	0.038 (10)	−0.004 (13)	0.017 (8)	−0.021 (13)
C106	0.027 (8)	0.028 (9)	0.145 (19)	−0.014 (7)	0.006 (10)	0.032 (10)
Cl1	0.128 (3)	0.0528 (17)	0.372 (7)	0.0370 (18)	0.190 (4)	0.051 (3)
Cl2	0.103 (2)	0.0839 (18)	0.0588 (15)	−0.0530 (16)	0.0399 (14)	−0.0097 (13)
C100	0.159 (12)	0.057 (6)	0.087 (8)	−0.038 (7)	0.083 (8)	−0.014 (6)
Br1	0.0423 (4)	0.0485 (5)	0.0337 (4)	0.0147 (4)	−0.0046 (3)	−0.0042 (4)
Br2	0.0248 (3)	0.0341 (4)	0.0367 (4)	−0.0028 (3)	0.0085 (3)	−0.0162 (3)
O1	0.065 (4)	0.025 (3)	0.036 (3)	−0.009 (2)	0.010 (3)	−0.001 (2)

### Geometric parameters (Å, º)

**Table:**

Ru1—P1	2.2857 (15)	C35—C36	1.396 (8)
Ru1—P2	2.2813 (14)	C36—C37	1.392 (9)
Ru1—N1	2.147 (4)	C37—C38	1.383 (9)
Ru1—N2	2.089 (5)	C38—C39	1.378 (8)
Ru1—N3	2.150 (5)	C40—C41	1.392 (7)
Ru1—N4	2.100 (4)	C40—C45	1.404 (7)
Cl3—C106	1.793 (16)	C41—C42	1.384 (8)
Cl4—C106	1.899 (15)	C42—C43	1.382 (7)
Cl4—C105	1.85 (2)	C43—C44	1.389 (9)
Cl7—C105	1.78 (2)	C44—C45	1.382 (8)
Cl1—C100	1.735 (13)	C46—C47	1.394 (7)
Cl2—C100	1.755 (11)	C46—C51	1.402 (7)
P1—C28	1.820 (5)	C47—C48	1.396 (7)
P1—C34	1.829 (6)	C48—C49	1.373 (8)
P1—N6	1.772 (5)	C49—C50	1.390 (9)
P2—C46	1.829 (5)	C50—C51	1.382 (7)
P2—N5	1.766 (5)	C2—H2B	0.9900
P2—C40	1.828 (5)	C2—H2A	0.9900
P4—F4	1.601 (4)	C3—H3B	0.9900
P4—F5	1.608 (4)	C3—H3A	0.9900
P4—F6	1.602 (4)	C4—H4A	0.9900
P4—F3	1.607 (4)	C4—H4B	0.9900
P4—F2	1.594 (4)	C5—H5B	0.9900
P4—F1	1.583 (4)	C5—H5A	0.9900
N1—C17	1.345 (6)	C6—H6A	0.9900
N1—C13	1.355 (6)	C6—H6B	0.9900
N2—C8	1.358 (7)	C7—H7A	0.9900
N2—C12	1.363 (7)	C7—H7B	0.9900
N3—C18	1.352 (7)	C8—H8	0.9500
N3—C22	1.367 (7)	C9—H9	0.9500
N4—C23	1.355 (7)	C10—H10	0.9500
N4—C27	1.352 (7)	C11—H11	0.9500
N5—C2	1.490 (7)	C14—H14	0.9500
N5—C1	1.375 (6)	C15—H15	0.9500
N6—C7	1.495 (6)	C16—H16	0.9500
N6—C1	1.365 (7)	C17—H17	0.9500
N7—C1	1.318 (6)	C18—H18	0.9500
N7—C4	1.482 (7)	C19—H19	0.9500
N7—C5	1.485 (6)	C20—H20	0.9500
O1—H1A	0.99 (5)	C21—H21	0.9500
O1—H1B	1.00 (4)	C24—H24	0.9500
C2—C3	1.512 (8)	C25—H25	0.9500
C3—C4	1.522 (9)	C26—H26	0.9500
C5—C6	1.524 (7)	C27—H27	0.9500
C6—C7	1.508 (8)	C29—H29	0.9500
C8—C9	1.371 (8)	C30—H30	0.9500
C9—C10	1.383 (9)	C31—H31	0.9500
C10—C11	1.384 (9)	C32—H32	0.9500
C11—C12	1.382 (8)	C33—H33	0.9500
C12—C13	1.471 (9)	C35—H35	0.9500
C13—C14	1.391 (8)	C36—H36	0.9500
C14—C15	1.378 (9)	C37—H37	0.9500
C15—C16	1.383 (7)	C38—H38	0.9500
C16—C17	1.387 (6)	C39—H39	0.9500
C18—C19	1.386 (8)	C41—H41	0.9500
C19—C20	1.385 (8)	C42—H42	0.9500
C20—C21	1.383 (7)	C43—H43	0.9500
C21—C22	1.393 (8)	C44—H44	0.9500
C22—C23	1.478 (7)	C45—H45	0.9500
C23—C24	1.390 (7)	C47—H47	0.9500
C24—C25	1.389 (8)	C48—H48	0.9500
C25—C26	1.381 (8)	C49—H49	0.9500
C26—C27	1.382 (7)	C50—H50	0.9500
C28—C29	1.394 (7)	C51—H51	0.9500
C28—C33	1.416 (8)	C105—H998	0.9600
C29—C30	1.397 (7)	C105—H999	0.9600
C30—C31	1.382 (9)	C106—H10D	0.9900
C31—C32	1.386 (9)	C106—H10C	0.9900
C32—C33	1.372 (8)	C100—H10B	0.9900
C34—C35	1.385 (8)	C100—H10A	0.9900
C34—C39	1.397 (9)		
			
P1—Ru1—P2	86.93 (5)	C47—C46—C51	118.5 (5)
P1—Ru1—N1	174.37 (10)	P2—C46—C47	126.2 (4)
P1—Ru1—N2	98.52 (12)	C46—C47—C48	119.9 (5)
P1—Ru1—N3	98.45 (12)	C47—C48—C49	120.9 (6)
P1—Ru1—N4	89.92 (12)	C48—C49—C50	119.8 (5)
P2—Ru1—N1	97.07 (10)	C49—C50—C51	119.7 (5)
P2—Ru1—N2	89.82 (14)	C46—C51—C50	121.2 (5)
P2—Ru1—N3	173.86 (12)	C3—C2—H2A	110.00
P2—Ru1—N4	99.68 (14)	N5—C2—H2A	110.00
N1—Ru1—N2	77.60 (15)	H2A—C2—H2B	108.00
N1—Ru1—N3	77.78 (15)	N5—C2—H2B	110.00
N1—Ru1—N4	93.30 (15)	C3—C2—H2B	110.00
N2—Ru1—N3	92.28 (19)	C2—C3—H3B	110.00
N2—Ru1—N4	167.64 (18)	C2—C3—H3A	110.00
N3—Ru1—N4	77.48 (19)	C4—C3—H3B	110.00
C105—Cl4—C106	172.2 (9)	C4—C3—H3A	110.00
Ru1—P1—C28	114.4 (2)	H3A—C3—H3B	108.00
Ru1—P1—C34	121.5 (2)	N7—C4—H4B	109.00
N6—P1—C28	106.0 (2)	C3—C4—H4A	109.00
N6—P1—C34	100.8 (2)	C3—C4—H4B	109.00
C28—P1—C34	99.5 (3)	H4A—C4—H4B	108.00
Ru1—P1—N6	112.58 (16)	N7—C4—H4A	109.00
Ru1—P2—N5	113.64 (17)	N7—C5—H5A	109.00
Ru1—P2—C46	114.31 (17)	N7—C5—H5B	109.00
N5—P2—C40	101.3 (2)	C6—C5—H5B	109.00
N5—P2—C46	105.4 (2)	H5A—C5—H5B	108.00
C40—P2—C46	99.1 (2)	C6—C5—H5A	109.00
Ru1—P2—C40	120.86 (16)	C5—C6—H6B	110.00
F4—P4—F6	89.75 (19)	C7—C6—H6A	110.00
F5—P4—F6	89.60 (19)	H6A—C6—H6B	108.00
F1—P4—F3	90.6 (2)	C7—C6—H6B	110.00
F1—P4—F4	179.0 (2)	C5—C6—H6A	110.00
F1—P4—F5	90.3 (2)	N6—C7—H7B	110.00
F1—P4—F6	89.9 (2)	C6—C7—H7A	110.00
F2—P4—F3	90.26 (18)	H7A—C7—H7B	108.00
F2—P4—F4	89.3 (2)	C6—C7—H7B	110.00
F2—P4—F5	178.0 (2)	N6—C7—H7A	110.00
F2—P4—F6	90.18 (19)	C9—C8—H8	119.00
F3—P4—F4	89.83 (18)	N2—C8—H8	119.00
F3—P4—F5	89.95 (18)	C10—C9—H9	120.00
F3—P4—F6	179.4 (2)	C8—C9—H9	121.00
F4—P4—F5	88.8 (2)	C9—C10—H10	120.00
F1—P4—F2	91.6 (2)	C11—C10—H10	120.00
Ru1—N1—C13	112.7 (4)	C12—C11—H11	120.00
Ru1—N1—C17	126.3 (3)	C10—C11—H11	120.00
C13—N1—C17	118.7 (4)	C13—C14—H14	120.00
Ru1—N2—C12	115.0 (3)	C15—C14—H14	120.00
Ru1—N2—C8	127.2 (4)	C14—C15—H15	120.00
C8—N2—C12	117.8 (5)	C16—C15—H15	120.00
C18—N3—C22	118.4 (5)	C15—C16—H16	121.00
Ru1—N3—C18	126.2 (4)	C17—C16—H16	121.00
Ru1—N3—C22	113.4 (4)	C16—C17—H17	119.00
C23—N4—C27	117.3 (4)	N1—C17—H17	119.00
Ru1—N4—C23	115.7 (3)	N3—C18—H18	119.00
Ru1—N4—C27	127.0 (3)	C19—C18—H18	119.00
C1—N5—C2	113.8 (4)	C18—C19—H19	121.00
P2—N5—C1	118.7 (4)	C20—C19—H19	121.00
P2—N5—C2	124.3 (4)	C21—C20—H20	120.00
C1—N6—C7	114.3 (4)	C19—C20—H20	120.00
P1—N6—C1	120.0 (3)	C20—C21—H21	120.00
P1—N6—C7	123.9 (4)	C22—C21—H21	121.00
C1—N7—C5	124.1 (5)	C25—C24—H24	120.00
C4—N7—C5	111.4 (4)	C23—C24—H24	120.00
C1—N7—C4	124.5 (4)	C26—C25—H25	121.00
H1A—O1—H1B	111 (4)	C24—C25—H25	121.00
N6—C1—N7	120.6 (4)	C27—C26—H26	120.00
N5—C1—N6	119.2 (4)	C25—C26—H26	120.00
N5—C1—N7	120.2 (5)	C26—C27—H27	118.00
N5—C2—C3	108.7 (4)	N4—C27—H27	118.00
C2—C3—C4	108.3 (4)	C28—C29—H29	120.00
N7—C4—C3	112.9 (4)	C30—C29—H29	120.00
N7—C5—C6	112.6 (4)	C31—C30—H30	120.00
C5—C6—C7	107.9 (5)	C29—C30—H30	120.00
N6—C7—C6	108.3 (4)	C30—C31—H31	120.00
N2—C8—C9	122.9 (5)	C32—C31—H31	120.00
C8—C9—C10	119.0 (6)	C33—C32—H32	120.00
C9—C10—C11	119.2 (6)	C31—C32—H32	120.00
C10—C11—C12	119.5 (6)	C28—C33—H33	120.00
N2—C12—C11	121.6 (6)	C32—C33—H33	120.00
N2—C12—C13	115.8 (4)	C34—C35—H35	120.00
C11—C12—C13	122.3 (5)	C36—C35—H35	120.00
N1—C13—C14	121.3 (5)	C37—C36—H36	120.00
C12—C13—C14	123.2 (5)	C35—C36—H36	120.00
N1—C13—C12	115.5 (5)	C38—C37—H37	120.00
C13—C14—C15	119.4 (5)	C36—C37—H37	120.00
C14—C15—C16	119.5 (5)	C37—C38—H38	120.00
C15—C16—C17	118.6 (4)	C39—C38—H38	120.00
N1—C17—C16	122.4 (4)	C38—C39—H39	120.00
N3—C18—C19	123.0 (5)	C34—C39—H39	120.00
C18—C19—C20	118.2 (5)	C40—C41—H41	119.00
C19—C20—C21	120.1 (5)	C42—C41—H41	119.00
C20—C21—C22	119.1 (5)	C43—C42—H42	120.00
N3—C22—C21	121.3 (5)	C41—C42—H42	120.00
C21—C22—C23	123.3 (5)	C44—C43—H43	120.00
N3—C22—C23	115.4 (5)	C42—C43—H43	120.00
N4—C23—C24	122.5 (4)	C45—C44—H44	120.00
C22—C23—C24	121.5 (5)	C43—C44—H44	120.00
N4—C23—C22	115.9 (5)	C40—C45—H45	120.00
C23—C24—C25	119.1 (5)	C44—C45—H45	119.00
C24—C25—C26	118.7 (5)	C48—C47—H47	120.00
C25—C26—C27	119.1 (5)	C46—C47—H47	120.00
N4—C27—C26	123.1 (4)	C47—C48—H48	119.00
C29—C28—C33	118.8 (5)	C49—C48—H48	120.00
P1—C28—C33	114.2 (4)	C50—C49—H49	120.00
P1—C28—C29	126.9 (4)	C48—C49—H49	120.00
C28—C29—C30	119.9 (5)	C49—C50—H50	120.00
C29—C30—C31	120.0 (6)	C51—C50—H50	120.00
C30—C31—C32	120.9 (6)	C50—C51—H51	119.00
C31—C32—C33	119.5 (6)	C46—C51—H51	119.00
C28—C33—C32	120.9 (5)	Cl4—C105—Cl7	109.7 (9)
P1—C34—C39	119.1 (4)	Cl3—C106—Cl4	109.9 (9)
P1—C34—C35	122.0 (5)	Cl4—C105—H998	112.00
C35—C34—C39	118.9 (5)	Cl7—C105—H998	109.00
C34—C35—C36	120.5 (6)	Cl7—C105—H999	108.00
C35—C36—C37	119.8 (6)	H998—C105—H999	107.00
C36—C37—C38	119.5 (6)	Cl4—C105—H999	111.00
C37—C38—C39	120.6 (6)	Cl3—C106—H10C	110.00
C34—C39—C38	120.6 (6)	Cl4—C106—H10C	110.00
P2—C40—C41	122.8 (4)	Cl4—C106—H10D	110.00
C41—C40—C45	117.8 (5)	Cl3—C106—H10D	110.00
P2—C40—C45	119.3 (4)	H10C—C106—H10D	108.00
C40—C41—C42	121.3 (5)	Cl1—C100—Cl2	111.5 (7)
C41—C42—C43	120.0 (5)	Cl1—C100—H10A	109.00
C42—C43—C44	119.9 (5)	Cl1—C100—H10B	109.00
C43—C44—C45	120.0 (5)	Cl2—C100—H10B	109.00
C40—C45—C44	121.0 (5)	H10A—C100—H10B	108.00
P2—C46—C51	115.3 (4)	Cl2—C100—H10A	109.00
			
P2—Ru1—P1—N6	26.11 (18)	C12—N2—C8—C9	1.1 (8)
N2—Ru1—P1—N6	−63.3 (2)	Ru1—N2—C12—C11	179.2 (4)
N3—Ru1—P1—N6	−156.9 (2)	Ru1—N2—C8—C9	179.2 (4)
N4—Ru1—P1—N6	125.8 (2)	Ru1—N2—C12—C13	−6.0 (6)
P2—Ru1—P1—C28	−95.00 (19)	Ru1—N3—C18—C19	163.8 (4)
N2—Ru1—P1—C28	175.6 (2)	C18—N3—C22—C23	179.0 (5)
N3—Ru1—P1—C28	82.0 (2)	Ru1—N3—C22—C23	14.1 (6)
N4—Ru1—P1—C28	4.7 (2)	C22—N3—C18—C19	1.0 (8)
P2—Ru1—P1—C34	145.6 (2)	Ru1—N3—C22—C21	−166.2 (4)
N2—Ru1—P1—C34	56.2 (3)	C18—N3—C22—C21	−1.2 (8)
N3—Ru1—P1—C34	−37.4 (3)	C27—N4—C23—C22	173.7 (5)
N4—Ru1—P1—C34	−114.7 (3)	C27—N4—C23—C24	−2.3 (8)
P1—Ru1—P2—N5	22.53 (17)	Ru1—N4—C23—C24	178.2 (4)
N1—Ru1—P2—N5	−161.45 (19)	Ru1—N4—C23—C22	−5.8 (6)
N2—Ru1—P2—N5	121.1 (2)	Ru1—N4—C27—C26	−179.4 (4)
N4—Ru1—P2—N5	−66.9 (2)	C23—N4—C27—C26	1.2 (7)
P1—Ru1—P2—C40	143.22 (19)	C2—N5—C1—N7	19.4 (7)
N1—Ru1—P2—C40	−40.8 (2)	C2—N5—C1—N6	−161.0 (5)
N2—Ru1—P2—C40	−118.2 (2)	P2—N5—C1—N6	38.0 (6)
N4—Ru1—P2—C40	53.8 (2)	P2—N5—C1—N7	−141.6 (4)
P1—Ru1—P2—C46	−98.57 (16)	C1—N5—C2—C3	−56.9 (6)
N1—Ru1—P2—C46	77.45 (19)	P2—N5—C2—C3	102.9 (5)
N2—Ru1—P2—C46	0.0 (2)	C1—N6—C7—C6	−55.9 (6)
N4—Ru1—P2—C46	172.04 (19)	P1—N6—C7—C6	108.5 (4)
P2—Ru1—N1—C13	−104.8 (3)	C7—N6—C1—N5	−162.4 (4)
N2—Ru1—N1—C13	−16.6 (3)	C7—N6—C1—N7	17.2 (7)
N3—Ru1—N1—C13	78.6 (3)	P1—N6—C1—N5	32.6 (6)
N4—Ru1—N1—C13	155.0 (3)	P1—N6—C1—N7	−147.8 (4)
P2—Ru1—N1—C17	92.7 (4)	C1—N7—C5—C6	−4.3 (8)
N2—Ru1—N1—C17	−179.1 (4)	C4—N7—C5—C6	176.2 (5)
N3—Ru1—N1—C17	−84.0 (4)	C5—N7—C4—C3	173.7 (4)
N4—Ru1—N1—C17	−7.5 (4)	C4—N7—C1—N5	13.1 (8)
P1—Ru1—N2—C8	18.0 (5)	C4—N7—C1—N6	−166.5 (5)
P2—Ru1—N2—C8	−68.9 (5)	C1—N7—C4—C3	−5.9 (7)
N1—Ru1—N2—C8	−166.2 (5)	C5—N7—C1—N5	−166.5 (5)
N3—Ru1—N2—C8	116.9 (5)	C5—N7—C1—N6	14.0 (8)
P1—Ru1—N2—C12	−163.9 (4)	N5—C2—C3—C4	60.8 (5)
P2—Ru1—N2—C12	109.2 (4)	C2—C3—C4—N7	−31.1 (5)
N1—Ru1—N2—C12	12.0 (4)	N7—C5—C6—C7	−33.7 (7)
N3—Ru1—N2—C12	−65.0 (4)	C5—C6—C7—N6	62.1 (6)
P1—Ru1—N3—C18	95.4 (4)	N2—C8—C9—C10	1.5 (9)
N1—Ru1—N3—C18	−80.3 (5)	C8—C9—C10—C11	−2.6 (11)
N2—Ru1—N3—C18	−3.5 (5)	C9—C10—C11—C12	1.3 (11)
N4—Ru1—N3—C18	−176.5 (5)	C10—C11—C12—N2	1.4 (9)
P1—Ru1—N3—C22	−101.1 (4)	C10—C11—C12—C13	−173.1 (6)
N1—Ru1—N3—C22	83.2 (4)	C11—C12—C13—N1	166.0 (5)
N2—Ru1—N3—C22	160.0 (4)	N2—C12—C13—N1	−8.8 (7)
N4—Ru1—N3—C22	−13.1 (4)	C11—C12—C13—C14	−15.7 (8)
P1—Ru1—N4—C23	108.8 (4)	N2—C12—C13—C14	169.5 (5)
P2—Ru1—N4—C23	−164.4 (4)	C12—C13—C14—C15	178.5 (5)
N1—Ru1—N4—C23	−66.6 (4)	N1—C13—C14—C15	−3.3 (7)
N3—Ru1—N4—C23	10.1 (4)	C13—C14—C15—C16	0.1 (7)
P1—Ru1—N4—C27	−70.7 (4)	C14—C15—C16—C17	2.1 (7)
P2—Ru1—N4—C27	16.2 (4)	C15—C16—C17—N1	−1.2 (7)
N1—Ru1—N4—C27	113.9 (4)	N3—C18—C19—C20	−0.6 (9)
N3—Ru1—N4—C27	−169.4 (4)	C18—C19—C20—C21	0.4 (8)
C34—P1—C28—C33	64.6 (5)	C19—C20—C21—C22	−0.6 (9)
C34—P1—N6—C1	162.1 (4)	C20—C21—C22—C23	−179.2 (5)
Ru1—P1—N6—C7	129.5 (4)	C20—C21—C22—N3	1.1 (8)
C28—P1—N6—C7	−104.7 (4)	C21—C22—C23—C24	−9.5 (9)
C34—P1—N6—C7	−1.4 (5)	N3—C22—C23—C24	170.2 (5)
N6—P1—C34—C39	−75.5 (5)	C21—C22—C23—N4	174.4 (5)
Ru1—P1—N6—C1	−67.0 (4)	N3—C22—C23—N4	−5.8 (7)
C28—P1—N6—C1	58.8 (5)	N4—C23—C24—C25	0.5 (9)
Ru1—P1—C28—C29	109.9 (5)	C22—C23—C24—C25	−175.3 (6)
N6—P1—C28—C29	−14.8 (6)	C23—C24—C25—C26	2.4 (9)
Ru1—P1—C34—C35	−20.2 (6)	C24—C25—C26—C27	−3.5 (9)
N6—P1—C28—C33	168.7 (4)	C25—C26—C27—N4	1.7 (8)
Ru1—P1—C28—C33	−66.6 (5)	P1—C28—C29—C30	−173.8 (5)
C28—P1—C34—C39	33.0 (5)	P1—C28—C33—C32	173.6 (5)
C34—P1—C28—C29	−119.0 (6)	C29—C28—C33—C32	−3.2 (9)
C28—P1—C34—C35	−146.7 (5)	C33—C28—C29—C30	2.6 (9)
N6—P1—C34—C35	104.9 (5)	C28—C29—C30—C31	−0.6 (9)
Ru1—P1—C34—C39	159.4 (4)	C29—C30—C31—C32	−0.8 (10)
C40—P2—N5—C1	162.1 (4)	C30—C31—C32—C33	0.2 (10)
C46—P2—N5—C1	59.2 (5)	C31—C32—C33—C28	1.8 (9)
Ru1—P2—N5—C2	134.4 (4)	C39—C34—C35—C36	−0.2 (9)
Ru1—P2—N5—C1	−66.8 (4)	P1—C34—C35—C36	179.5 (5)
N5—P2—C40—C41	103.4 (4)	C35—C34—C39—C38	−1.7 (9)
N5—P2—C46—C47	−12.1 (5)	P1—C34—C39—C38	178.7 (5)
C40—P2—N5—C2	3.2 (5)	C34—C35—C36—C37	2.3 (9)
Ru1—P2—C40—C41	−23.2 (5)	C35—C36—C37—C38	−2.5 (10)
C46—P2—N5—C2	−99.7 (4)	C36—C37—C38—C39	0.7 (9)
C40—P2—C46—C47	−116.6 (5)	C37—C38—C39—C34	1.4 (9)
C46—P2—C40—C41	−148.8 (4)	P2—C40—C41—C42	177.8 (4)
C46—P2—C40—C45	28.6 (4)	C41—C40—C45—C44	−0.5 (8)
C40—P2—C46—C51	63.6 (4)	C45—C40—C41—C42	0.4 (7)
Ru1—P2—C46—C51	−66.4 (4)	P2—C40—C45—C44	−178.0 (4)
Ru1—P2—C46—C47	113.4 (4)	C40—C41—C42—C43	−0.8 (8)
N5—P2—C40—C45	−79.3 (4)	C41—C42—C43—C44	1.3 (8)
N5—P2—C46—C51	168.1 (4)	C42—C43—C44—C45	−1.3 (9)
Ru1—P2—C40—C45	154.2 (3)	C43—C44—C45—C40	1.0 (9)
Ru1—N1—C13—C12	18.6 (5)	P2—C46—C47—C48	−179.7 (4)
C13—N1—C17—C16	−1.9 (7)	C51—C46—C47—C48	0.2 (7)
Ru1—N1—C17—C16	159.7 (4)	P2—C46—C51—C50	178.8 (4)
Ru1—N1—C13—C14	−159.8 (4)	C47—C46—C51—C50	−1.1 (8)
C17—N1—C13—C14	4.2 (7)	C46—C47—C48—C49	0.7 (8)
C17—N1—C13—C12	−177.5 (4)	C47—C48—C49—C50	−0.7 (8)
C8—N2—C12—C11	−2.5 (8)	C48—C49—C50—C51	−0.2 (8)
C8—N2—C12—C13	172.3 (5)	C49—C50—C51—C46	1.1 (8)

### Hydrogen-bond geometry (Å, º)

**Table:**

*D*—H···*A*	*D*—H	H···*A*	*D*···*A*	*D*—H···*A*
O1—H1*A*···Br1^i^	0.99 (5)	2.31 (5)	3.300 (5)	173 (3)
O1—H1*B*···Br1^ii^	1.00 (4)	2.35 (4)	3.345 (5)	178 (6)
C4—H4*A*···Br2^iii^	0.99	2.90	3.651 (5)	133
C6—H6*A*···Cl7	0.99	2.82	3.738 (8)	155
C6—H6*B*···O1^iv^	0.99	2.52	3.358 (8)	142
C9—H9···O1^v^	0.95	2.40	3.208 (8)	142
C10—H10···Cl3^vi^	0.95	2.72	3.316 (8)	122
C100—H10*A*···Br1^vii^	0.99	2.92	3.618 (11)	128
C106—H10*C*···Cl1	0.99	2.65	3.54 (2)	150
C106—H10*D*···O1^iv^	0.99	2.51	3.464 (16)	162
C11—H11···F3^vii^	0.95	2.35	3.209 (7)	150
C17—H17···F5^iii^	0.95	2.25	3.056 (6)	142
C21—H21···Br1^viii^	0.95	2.91	3.787 (5)	154
C24—H24···Br1^viii^	0.95	2.91	3.835 (5)	164
C25—H25···F4^ix^	0.95	2.54	3.453 (6)	160
C31—H31···Cl3^x^	0.95	2.76	3.566 (7)	143
C42—H42···F3^iii^	0.95	2.41	3.319 (7)	160

Symmetry codes: (i) −*x*+3/2, *y*+1/2, −*z*+1/2; (ii) *x*+1/2, −*y*+1/2, *z*−1/2; (iii) −*x*+1/2, *y*−1/2, −*z*+3/2; (iv) −*x*+1, −*y*+1, −*z*+1; (v) *x*−1, *y*, *z*+1; (vi) −*x*, −*y*+1, −*z*+2; (vii) *x*−1/2, −*y*+1/2, *z*+1/2; (viii) *x*, *y*, *z*+1; (ix) *x*+1/2, −*y*+1/2, *z*+1/2; (x) −*x*+1, −*y*+1, −*z*+2.

## References

[bb1] Agilent (2012). *CrysAlis PRO* Agilent Technologies Ltd, Yarnton, England.

[bb2] Altomare, A., Cascarano, G., Giacovazzo, C., Guagliardi, A., Burla, M. C., Polidori, G. & Camalli, M. (1994). J. Appl. Cryst. 27, 435.

[bb3] Farrugia, L. J. (2012). *J. Appl. Cryst.* **45**, 849–854.

[bb4] Sheldrick, G. M. (2008). *Acta Cryst.* A**64**, 112–122. 10.1107/S0108767307043930 18156677

[bb5] Spek, A. L. (2009). *Acta Cryst.* D**65**, 148–155. 10.1107/S090744490804362X PMC2631630 19171970

[bb6] Sullivan, B. P., Salmon, D. J. & Meyer, T. J. (1978). *Inorg. Chem* **17**, 3334–3341.

